# Discovery and Design of Novel Small Molecule GSK-3 Inhibitors Targeting the Substrate Binding Site

**DOI:** 10.3390/ijms21228709

**Published:** 2020-11-18

**Authors:** Ido Rippin, Netaly Khazanov, Shirley Ben Joseph, Tania Kudinov, Eva Berent, Sara Melisa Arciniegas Ruiz, Daniele Marciano, Laura Levy, Arie Gruzman, Hanoch Senderowitz, Hagit Eldar-Finkelman

**Affiliations:** 1 Department of Human Molecular Genetics & Biochemistry, Sackler School of Medicine, Tel Aviv University, Tel Aviv 6997801, Israel; idorip.ny@gmail.com (I.R.); shirley_joseph@walla.co.il (S.B.J.); tania.altman@gmail.com (T.K.); evabr@tauex.tau.ac.il (E.B.); smarciniegasr@gmail.com (S.M.A.R.); 2 Department of Chemistry, Bar-Ilan University, Ramat Gan 5290002, Israel; netalyk@gmail.com (N.K.); levylaura55@hotmail.com (L.L.); Aric-Lev.Gruzman@biu.ac.il (A.G.); hsenderowitz@gmail.com (H.S.); 3 Israel Institute for Biological Research, Ness Ziona 7410001, Israel; daniele.marciano@gmail.com

**Keywords:** GSK-3, pharmacophore, virtual screening, small molecules, substrate competitive inhibitors, peptides

## Abstract

The serine/threonine kinase, GSK-3, is a promising drug discovery target for treating multiple pathological disorders. Most GSK-3 inhibitors that were developed function as ATP competitive inhibitors, with typical limitations in specificity, safety and drug-induced resistance. In contrast, substrate competitive inhibitors (SCIs), are considered highly selective, and more suitable for clinical practice. The development of SCIs has been largely neglected in the past because the ambiguous, undefined nature of the substrate-binding site makes them difficult to design. In this study, we used our previously described structural models of GSK-3 bound to SCI peptides, to design a pharmacophore model and to virtually screen the “drug-like” Zinc database (~6.3 million compounds). We identified leading hits that interact with critical binding elements in the GSK-3 substrate binding site and are chemically distinct from known GSK-3 inhibitors. Accordingly, novel GSK-3 SCI compounds were designed and synthesized with IC_50_ values of~1–4 μM. Biological activity of the SCI compound was confirmed in cells and in primary neurons that showed increased β-catenin levels and reduced tau phosphorylation in response to compound treatment. We have generated a new type of small molecule GSK-3 inhibitors and propose to use this strategy to further develop SCIs for other protein kinases.

## 1. Introduction

Protein kinases (PK) are important regulators of many biological processes, and represent an important class of targets for a diversity of human diseases and pathologies [1,2,3]. Most protein kinase inhibitors developed to date are small molecules that compete with the ATP binding of the kinase. This type of inhibitor, although powerful, often has limited specificity [4,5] because the ATP binding site is highly conserved among protein kinases [2,3]. Indeed, the vast majority of these inhibitors interact and cross-react with multiple members of the PK family [4,5,6], furthermore, they tend to induce drug resistance due to the formation of point mutations at the ATP binding site [6,7,8]. Thus, a different type of inhibitors that do not target the ATP binding site may constitute reliable PKs inhibitors for clinical use.

A different class of PK inhibitors, although not extensively studied, represents the substrate competitive inhibitors (SCIs). SCIs interact with the less conserved (and consequently, more specific) substrate-binding cavity of the kinase [2,3] and hold great promise as new therapeutics because they are highly selective, considered safe, and are less prone to drug-induced resistance [9,10,11]. However, SCI development has been somewhat neglected in the past, particularly SCIs in the form of small molecules. Clearly, SCIs were considered difficult to design due to the ambiguous, non-well-defined substrate binding site which is usually large and shallow. Thus, the search and development of SCIs is a challenging task.

Target-based docking screens for novel ligands have been largely used over the last decades and became principal tools in drug discovery [12,13,14,15]. The prior knowledge of protein–ligand intermolecular interactions is indeed a key for the identification of reliable hits that will later serve as successful starting points for drug design. However, the scarce number of available structures of PKs bound to their substrates discouraged perusing in silico-based searches for PKs-SCI. Indeed, to the best of our knowledge, computational approaches have not been practiced for the identification of small molecules SCIs.

The serine-threonine kinase, GSK-3, is a validated target for drug discovery in treating several pathologies including diabetes, neuronal development, neurodegenerative diseases and psychiatric disorders [16,17,18,19,20,21]. In humans, GSK-3 is expressed as two isozymes, GSK-3α and GSK-3β, which are encoded by two genes and share high homology in their catalytic domains [22]). The mechanisms by which GSK-3 is thought to contribute to pathogenesis are diverse. These include phosphorylation of the microtubule-associated protein tau [23,24], destabilization of the Wnt signaling component β-catenin [25,26], regulation of multiple transcript factors such as NF-κB [27,28], activation of pro-inflammatory factors [29], and impairment of cellular clearance pathways [30,31]. Inhibition of GSK-3 was considered a promising therapeutic approach, however, GSK-3 ATP competitive inhibitors failed in the pre-clinical phase due to toxicity and side effects.

In previous works, we developed a series of short phosphorylated SCI peptides for GSK-3. The SCI peptides were derived from the unique substrate-recognition motif of GSK-3, S^1^XXXS^2^(p) (where S^1^ is the GSK-3-phosphorylation site and S^2^ is the phosphorylated priming site) [32,33] and are patterned after the GSK-3 substrate heat shock factor-1 (HSF-1) [34]). The combined computational and biochemical analysis identified the critical sites important for the GSK-3 SCI peptides interactions with the GSK-3 substrate-binding site [33,35]. GSK-3 binds the “primed” phosphorylated peptide inhibitor (that mimics the primed phosphorylated substrate) through its “phosphate-binding pocket” (Arg 96, Arg 180, and Lys 205) [36,37]. In addition, the substrate binds to a segment bordered by Gln 89-Asn 95, termed the “89–95” loop in which Phe 93 is the most critical residue for binding [33,35]. In some cases, the SCI peptides interact with a “hydrophobic patch” (Val 214, Ile 217, and Tyr 216) located in proximity to the enzyme’s phosphate-binding pocket [33]). The therapeutic potential of our SCI peptides was demonstrated in several cellular and disease mouse models including Parkinson’s, Alzheimer’s disease, multiple sclerosis, depressive behavior, and Fragile X syndrome [31,32,38,39,40,41,42]. Here we aimed to develop small molecule SCIs for GSK-3. Based on our structural GSK-3-SCI peptide-binding model we discovered and designed novel GSK-3 SCI molecules and confirmed their biological activity in vitro and in cellular systems.

## 2. Results

### 2.1. The Use of GSK-3/SCI Peptide Binding Model for Pharmacophore-Based Virtual Screening

**Figure 1:**
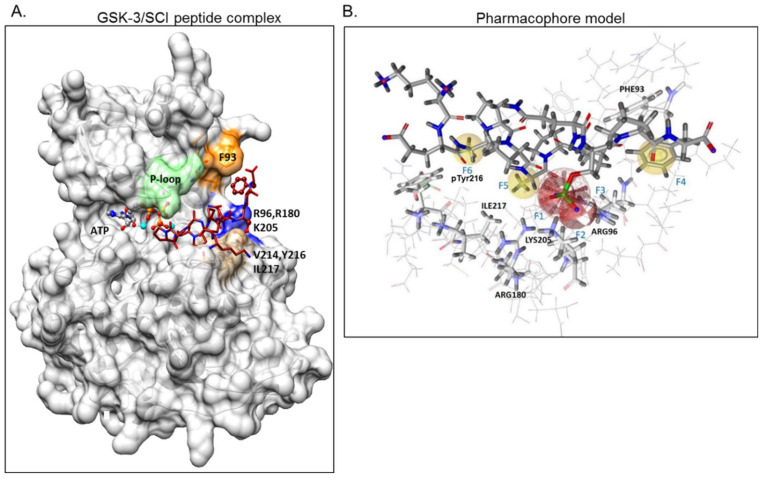
Design of a pharmacophore model based on GSK-3β binding model with substrate competitive inhibitor (SCI) peptide (**A**) Structural model of GSK-3β bound to our previously described SCI peptide L803F. The primed. phosphate S^10^(p) in the peptide interacts with the phosphate-binding pocket (Arg 96, Arg 180, Ly 205, marked blue), Phe^12^, at the C-terminal end of the peptide, interacts with Phe^93^ located in the “89–95” loop (orange), Ala^3^ in the peptide interacts with pTyr^216^, and Pro^5^ in the peptide interacts with Val ^214^ and Ile^217^, all residues that form the “hydrophobic patch” (beige). The P-loop including Phe^67^ is in green, the ATP molecule and Mg^+2^ (cyan balls) are shown. (**B**) The pharmacophore model is composed of two hydrogen bond acceptor features (F1, F2: red vectors), one anionic feature (F3: red porcupine shape), and three hydrophobic features (F4, F5, F6: yellow spheres). Excluded volumes were placed in positions that are sterically claimed by the protein environment. GSK-3 interacting residues are marked.

**Figure 2:**
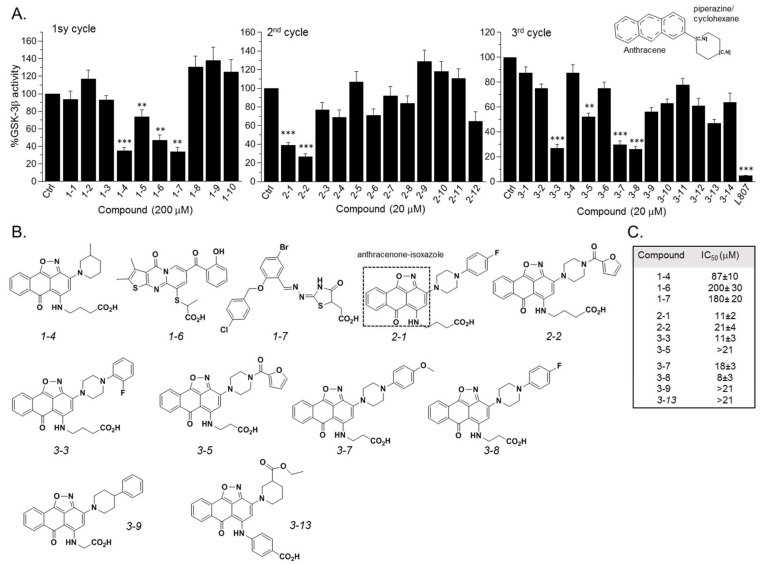
Discovery of GSK-3 SCI hits. (**A**) In vitro GSK-3β kinase assays were conducted with selected hits identified in the three iterative virtual screening cycles. Structure of compounds is summarized in Tables S1–S3. Ctrl represents GSK-3 activity no compounds (100%). Results are mean of three independent experiments ± SEM using one-way ANOVA with Dunnett’s post hoc test. ** *p* < 0.01 *** *p* < 0.001. The anthracene core used in the third search cycle is illustrated at the top right panel. The SCI peptide L807mts (L807) was used as a reference in the 3rd cycle assays. (**B**) Chemical structures of selected best hits of each cycle. The anthracenone–isoxazole core is highlighted in compound *2-1*. (**C**) IC_50_ values of selected best hits.

GSK-3β binding with the SCI peptide L803F (KEAPPSPPQS(p)PF) is shown in Figure 1A. This peptide is an improved version of our “original” L803 peptide inhibitor (KEAPPSPPQS(p)P) [33,43]. The model highlights the important features in substrate/SCI binding into the GSK-3β catalytic groove: the phosphorylated serine interacts with the phosphate-binding pocket of the kinase (Arg 96, Arg 180, and Lys 205) [36,37], prolines and phenylalanine in L803F interact with Phe 93, a part of the “89–95” loop, and a critical residue in substrate binding [33], the N-terminal end of L803F interacts with a “hydrophobic patch” in GSK-3β composed of Tyr 216, Val 214, and Ile 217 (Figure 1A) [33]. Thus, small molecules that mimic this binding mode should function as potent SCI molecules and we set out to discover such molecules. The similarity to *1-4*, *1-6*, and *1-7* (The hits 241 analogues for *1-4*, 11 analogues for *1-6*, and 21 analogues for *1-7*) were filtered as described above, and 12 available compounds were purchased (Table S2) and tested (Figure 2A). Compounds *2-1* and *2-2* (*1-4* analogues, Figure 2B) acted as best GSK-3β inhibitors (Figure 2A,C). As *1-4* analogues seemed more efficacious than the analogues related to *1-6* or *1-7*, we conducted a third search looking for molecules with the substructure core of *1-4*: anthracene attached to a piperazine/cyclohexane ring (see illustration, Figure 2A). We reasoned that suitable bioactive analogues could have been overlooked by our initial search, in addition, the fact that ZINC database is updated on a regular basis, and new compounds may become available. This search identified 137 hits, in which 15 were compatible with an interaction with the GSK-3β site, and 14 available compounds were purchased (Table S3). In vitro GSK-3 kinase assays indicated that most of the hit compounds acted as GSK-3β inhibitors (Figure 2A). The SCI peptide L807mts [32]) was used as a reference in these assays (Figure 2A 3rd cycle). Among them, *3-3*, *3-5*, *3-7*, *3-8*, *3-9* and *3-13* demonstrated best performance as GSK-3β inhibitors with IC_50_ values of ~8–20 μM. (Figure 2B,C).

### 2.2. SCI Hits Interact with the GSK-3β Substrate Binding Site and Are Chemically Unique 

**Figure 3:**
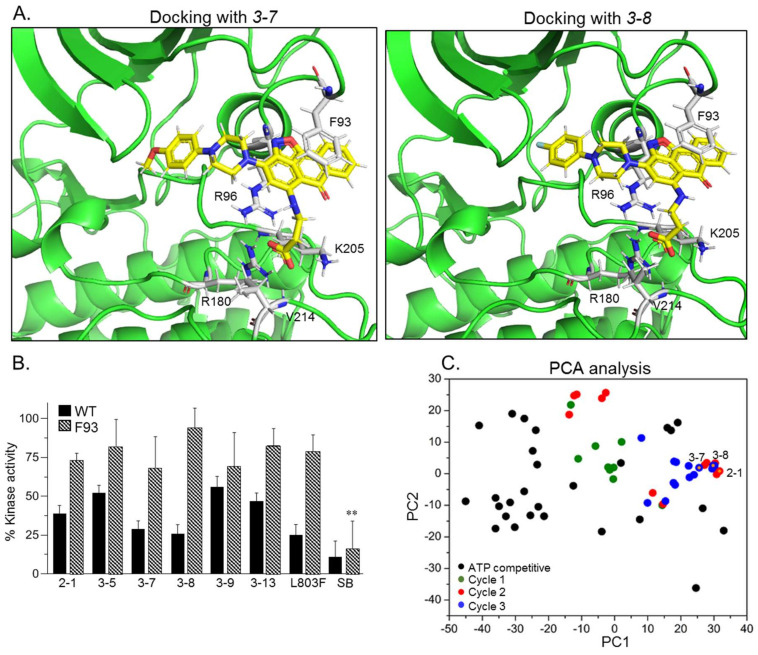
GSK-3 SCI compounds interact with the kinase substrate binding site and are chemically unique. (**A**) Docking models of compounds *3-7* and *3-8* with GSK-3β. Key interacting GSK-3β residues are highlighted, and detailed interactions are summarized in Table 1. The carboxylic acid moiety form salt bridges and H-bonding with the GSK-3-phosphate-binding pocket, Arg 96, Arg 180, Lys 205, and with Val 214. The anthracenone–isoxazole core forms π–π stacking interactions with Phe 93. Interactions were analyzed by the Maestro software. (**B**) In vitro kinase assays were performed with GSK-3β (WT) or with the F93A mutant in the presence of indicated compounds, L803F (20 μM each), and SB216763 (1 μM). Results are mean of three independent experiments ± SEM analyzed by one way ANOVA with Dunnett’s multiple comparisons. ** *p* < 0.01 with inhibitor vs no inhibitor (**C**) Principal component analysis (PCA) analysis of all compounds discovered through the three search cycles together with representative GSK-3-ATP competitive inhibitors (listed in Table S4). The first and second PCs accounted for 81.8% and 7.7% of the original variance and are shown at the *X-* and *Y*-axis respectively. Black circles represent the ATP competitive inhibitors, colored circles represent compounds from cycle 1 (green), 2 (red) and 3 (blue). Circles with yellow dots represent compounds *2-1*, *3-7* and *3-8*.

Docking analysis indeed showed that the identified compounds matched the pharmacophore requirements and shared similar structural features (Table 1). Representative docking models of GSK-3β with the best hits *3-7* and *3-8* are also shown in Figure 3A. Clearly, all discovered compounds shared a common carboxylic acid moiety that interacted with the phosphate-binding pocket (Arg 96, Arg 180, Lys 205) through salt bridges and H-bonds. This novel observation indicated for the first time that a non-phosphorylated group can mimic the phosphorylated substrates of GSK-3. In some cases, the carboxylic acid moiety formed H-bonds with Val 214, which also participates in GSK-3-L803F binding (Figure 1A) [33]. Most of the compounds contained an anthracenone–isoxazole core that formed π–π stacking and/or cation–π interactions with Phe 93 and Arg 96 respectively. In some instances, N or O at the isoxazol ring formed H-bonds with Arg 96, and the benzoic acid moieties interacted with Lys 85, Asn 95, and Glut-97, all important residues for kinase catalytic activity: Lys 85 and Glut 97 are highly conserved residues that facilitate interactions with ATP, and Asn 95 participate in substrate binding [33,44,45]. Take, together, our discovered compounds captured the binding mode of the GSK-3-SCI peptide complex.

To further confirm that compounds interact with the GSK-3β substrate binding site, we performed in vitro kinase assays with the GSK-3β-F93A mutant in which Phe 93 was replaced by alanine. This mutant is active but does not bind substrates or SCI peptides efficiently [33]. Indeed, like the SCI peptide L803F, F93A was barely inhibited by the SCI compounds (Figure 3B). This, in contrast, to SB216763, an established GSK-3 ATP competitive inhibitor [46] that inhibited both wild-type (WT) and the F93A mutant (Figure 3B). 

Finally, we conducted a principal component analysis (PCA) to determine whether the SCI compounds are chemically distinct from other known GSK-3 inhibitors. This was performed to confirm that the discovered compounds are unique and worthy of further development. Briefly, PCA projects a dataset originally described in a high-dimensional space into a 2-dimensional space while keeping, as much as possible, the original distribution of the data points (i.e., the distances between them). The analysis was performed on all SCI compounds (Tables S1–S3) together with representative ATP competitive GSK-3 inhibitors including SB-216763, CHIR98014, AR-A014418, VP2.51, Bio6, Kenpaullone, and 1-Azakenpaullone (listed in Table S4). The analysis clearly showed that the SCI compounds occupy a chemical space distinct from that occupied by the ATP competitive inhibitors (Figure 3C). The PCA analysis also revealed the successive focusing toward the active compounds region obtained through the three search cycles. These results encouraged further development of the discovered hits.

### 2.3. Design and Synthesis of Novel GSK-3 SCIs 

**Figure 4:**
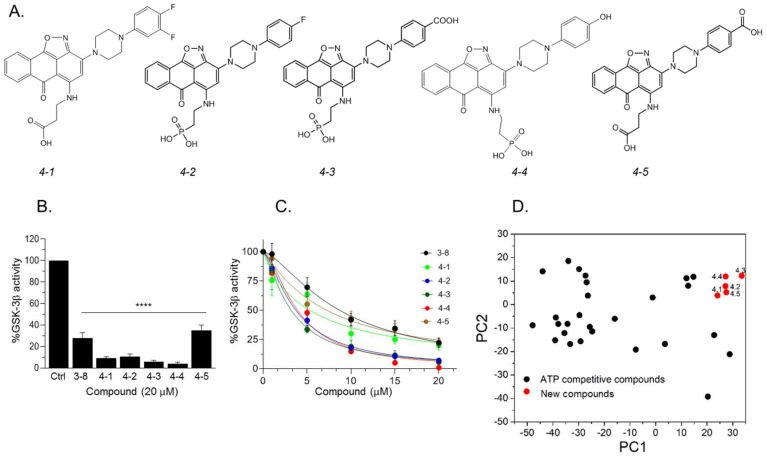
Newly designed GSK-3 SCI molecules. (**A**) Structures of new molecules *4-1* to *4-5*. (**B**) In vitro GSK-3β kinase assays were conducted with new compounds and *3-8* (20 μM each). Results show the percentage of GSK-3β activity without inhibitor (100%) and represent the mean of three independent experiments ± SEM analyzed by one way ANOVA with Dunnett’s multiple comparisons. **** *p* < 0.0001. (**C**) Dose–response curves of GSK-3β inhibition of new compounds and as compared to *3-8*. Results are mean of three independent experiments ± SEM. For *4-3* and *4-4* * *p* < 0.05 for all concentrations, for the rest of the molecules, * *p* < 0.05 at concentrations ≥ 5 μM as determined by one way ANOVA with Dunnett’s multiple comparisons, new compounds vs. *3-8*. (**D**) PCA analysis of new compounds *4-1* to *4-5* together with GSK-3-ATP competitive inhibitors (listed in Table S4). The first and second PCs accounted for 81.8% and 7.7% of the original variance and are shown at the *X*- and *Y*-axis respectively. Black circles represent the ATP competitive inhibitors, red circles represent new compounds.

Our results so far indicated that the discovered hits can represent a new class of GSK-3 inhibitors that act as SCIs. Based on the leading compounds *3-7* and *3-8*, we designed and synthesized new SCI molecules. We first attempted to replace the carboxylic acid moiety (CH_2_CO_2_H) with a phosphonate group (CH_2_PO_3_H_2_) to better mimics the “native” phosphorylation in GSK-3 substrates (GSK-3 substrate are pre-phosphorylated) [32,33]. In addition, we assumed that substituting the fluorine atom at the aromatic ring or other electron-withdrawing group such as CO_2_H, or, OH, or an additional fluorine atom to the ring, will facilitate additional contacts with the kinase. The newly synthesized compounds termed *4-1*, *4-2*, *4-3*, *4-4*, and *4-5* are presented in Figure 4A. In vitro kinase assays confirmed that the new compounds inhibited GSK-3β and were indeed better inhibitors as compared to *3*-8 (Figure 4B,C). Collectively, compounds *4-3* and *4-4* acted as best inhibitors showing IC_50_ values of ~1–4 μM. PCA analysis further confirmed that the new compounds are chemically distinct from other GSK-3 inhibitors (Figure 4D).

**Figure 5:**
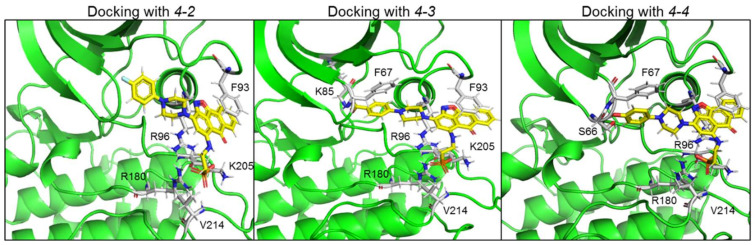
Docking new GSK-3 SCI molecules in GSK-3 substrate binding site. Docking models of GSK-3β bound to *4-2*, *4-3* and *4-4*. Detailed interactions are also summarized in Table 2. Like *3-8* and the other leading hits, the new molecules interacted with the GSK-3 phosphate-binding pocket, Phe 93 and Val 214. *4-3* and *4-4* showed additional interactions with Phe 67, Ly 85 (*4-3*), or, Ser 66 (*4-4*). Interactions were analyzed by the Maestro software.

Representative docking models of GSK-3β bound to *4-2*, *4-3* and *4-4* are shown in Figure 5, and detailed interactions are summarized in Table 2. The new compounds showed similar docking poses as those produced with *3-8*. As expected, the PO_3_H_2_ moiety interacted with the phosphate-binding pocket, and the anthracenone–isoxazole core formed π–π stacking interactions with Phe 93. Compounds *4-3* and *4-4* formed additional interactions with N or O of the isoxazole ring, and *4-4* formed cation–π interactions with Arg 96. In addition, *4-3* and *4-4* extended their interactions with Phe 67, Lys 85, or, Ser 66 through their CO_2_H or OH moieties respectively. These residues play a role in GSK-3-substrate binding [35,45]. Thus, the additional interactions performed with *4-3* and *4-4* may explain the improvement in their ability to inhibit GSK-3.

### 2.4. New Compounds Function as GSK-3 SCIs and Show Selectivity

**Figure 6:**
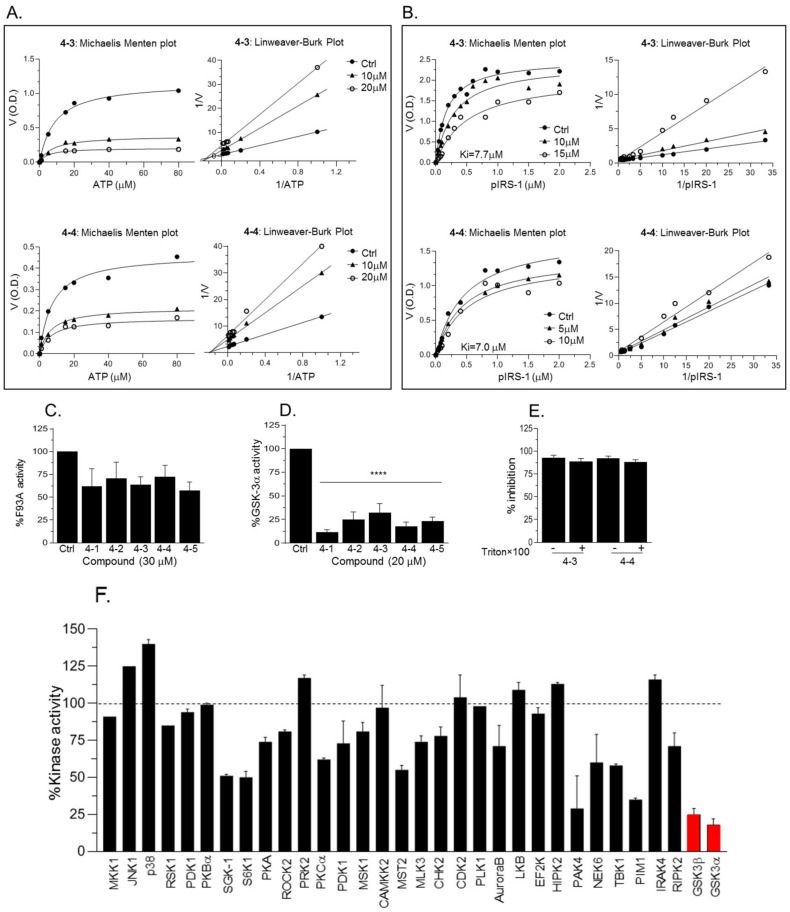
Kinetic analysis of new compounds. (**A**) Michaelis Menten plots of ATP competitive assays with *4-3* and *4-4.* The corresponding Lineaver–Burk plots are shown at the right panel. (**B**) Michaelis Menten plots of substrate competitive assays with *4-3* and *4-4.* The corresponding Lineaver–Burk plots are shown at the right panel. (**C**) In vitro kinase assays of F93A mutant in the presence of *4-1* through *4-5* (20 μM each). Results are mean of three independent experiments ± SEM using one-way ANOVA with Dunnett’s post hoc test. (**D**) GSK-3α kinase assays were performed with *4-1* through *4-5* (20 μM each). Results the mean of three independent experiments ± SEM using one-way ANOVA with Dunnett’s post hoc test. **** *p* < 0.0001 treated vs. control. (**E**) GSK-3β kinase assays were performed with *4-3* and *4-4* (20 μM) in the presence of 0.05% Triton × 100. (**F**) Kinase assays were performed with a representative repertoire of protein kinases in the presence of *4-4* (10 μM). Inhibition by *4-4* is presented as a percentage of kinase activity in the control assay with no inhibitor. Results are means ± SD of two independent experiments performed in duplicates. GSK-3α/β are marked red.

We next performed Michaelis Menten competitive assays to examine the ability of *4-3* or *4-4* to compete with ATP or with substrates. Michaelis Menten ATP competitive assays were performed and data were analyzed by Lineweaver–Burk plots indicating that compounds acted as non-competitive inhibitors for ATP (Figure 6A). Subsequently, Michaelis Menten substrate competitive assays were performed and data were analyzed by Lineweaver–Burk plots (Figure 6B). Indeed, respective lines intersected at the *Y*-axis, indicating that *4-3* and *4-4* function as substrate competitive inhibitors (Figure 6B). The calculated ki values were 7.7 ± 3 and 7.0 ± 0.2 for *4-3* and *4-4* respectively. We note that at high concentrations of the compounds (>20 μM) the Vamx decreased as compared to the control suggesting possible mixed inhibition under these conditions. Furthermore, the fact that *4-3* and *4-4* did not inhibit GSK-3β-F93A mutant (Figure 6C) provided additional support to our notion that *4-3* and *4-4* interact with the GSK-3 substrate binding site (Figure 6C). While most of our experiments concentrated on GSK-3β, we also tested the inhibition of GSK-3α by compounds *4-1* through *4-5*. We found that GSK-3α was indeed inhibited in a similar manner (Figure 6D). This result is not surprising as the catalytic domain of GSK-3α and GSK-3β share high similarity and the sites important for substrate’s binding are identical. Since small molecules tend to form colloidal aggregates that result in a non-specific aggregate-enzyme inhibition [47], we conducted GSK-3β kinase assays with increasing concentrations of Triton ×100 that was shown to disrupt colloidal aggregates [47]. Inhibition of GSK-3β by *4-3* or *4-4* was not affected by the detergent (Figure 6E). As a final caveat, we evaluated the selectivity of our leading compound *4-4*. Representative results of assays performed with 30 protein kinases (Figure 6F) demonstrated that *4-4* showed specificity toward GSK-3α and GSK-3β.

### 2.5. Biological Activity of the GSK-3 SCI Compounds

**Figure 7:**
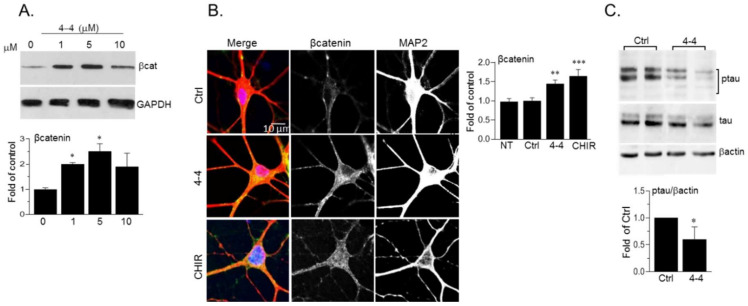
Biological activity of *4-4*. (**A**) SH-SY5Y cells were treated with *4-4* for 4 h and levels of cytoplasmic β-catenin were determined by immunoblot analysis. Densitometry analysis is shown on the lower panel. Results are mean of three independent experiments ± SEM * *p* < 0.05 by one-way ANOVA with Dunnett’s post hoc test (**B**) Hippocampal mouse neurons were treated with *4-4* (5 μM), or CHIR99021 (CHIR, 10 μM) for 4 h. Cells were co-stained with anti-β-catenin and anti-MAP2 antibodies. Images show overlapping β-catenin (green) and MAP2 (red) staining along with respective split channels (shown in grey). The β-catenin signal was evaluated by Image J Colocalization-finder plugin. Results present the mean of 60 cells ± SEM, ** *p* < 0.01, *** *p* < 0.0001 by one-way ANOVA with Dunnett’s post hoc test. NT- non treated. (**C**) Hippocampal mouse neurons were treated with *4-4* (20 μM) for 4 h. Levels of phosphorylated tau (Ser 396), tau, and β-actin were determined by immunoblot analysis. Bar graph represents densitometry analysis of ptau/βactin and results are mean of three independent experiments ± SEM using Student’s *t*-test. * *p* < 0.05. For all panels, Ctrl or 0 concentration represents cells treated with vehicle (DMSO/1%Tween 80 at matched dilutions—1:2000–4000).

To verify the potential biological activity of our SCI compounds, we tested their ability to inhibit cellular GSK-3. We focused on the SCI compound *4-4* that was the best inhibitor of the five molecules that were tested (*4-1*–*4-5*) β-catenin, which is a central Wnt signaling component, and a well-established GSK-3 target [25,48]. β-catenin phosphorylation by GSK-3 triggers its rapid proteasomal degradation [25,48]. We, therefore, studied potential alterations in β-catenin levels following treatment with *4-4*. Human neuroblastoma SH-SY5Y cells were treated with increasing concentrations of *4-4*, and the levels of β-catenin were determined in the cytoplasmic fraction (prepared after cell permeabilization and sup collection), this to avoid possible “masking” of membrane-associated β-catenin. The results indicated that treatment with *1-5* μM of *4-4* was sufficient to activate β-catenin in the cytoplasm (Figure 7A). The reproducible reduction in cytoplasmic β-catenin in response to higher doses (10 μM) is probably a consequence of β-catenin translocation to the nucleus [25] The compounds were not toxic to cells within the concentrations tested (1–20 μM) and at 24–72 hr post-treatment. As a last caveat, we tested *4-4* in neurons, which represent a “more relevant” physiological cell system. Mouse hippocampal primary neurons were prepared from 1-day old pups, and were treated with *4-4*, or, CHIR9920, a known ATP competitive inhibitor. The cells were then immunostained for β-catenin. A significant increase in β-catenin levels was detected in the soma and dendrites of neurons treated with *4-4* or CHIR9920 (Figure 7B). Phosphorylation of tau (Ser 396), a known GSK-3 substrate in neurons [23,24] was reduced following treatment with *4-4* as demonstrated by immunoblot analysis (Figure 7C). Together, these results indicated that *4-4* can inhibit cellular GSK-3.

## 3. Discussion

Despite the anticipated advantages of SCIs for protein kinases, only a few efforts were devoted to their development, likely due to the difficulty in their discovery and design. Here, we describe a rational strategy for the discovery and design of small molecule SCIs for GSK-3 and by extension, to other protein kinases. The work was based on SCI peptides that we previously developed, and was motivated by the need to overcome peptides limitations as therapeutics [49]. We assumed that the unique interactions formed between GSK-3 and its SCI peptides will provide a reliable template for the identification of the “correct” molecules that truly bind to the GSK-3 substrate binding site. To the best of our knowledge, these are the first kinase-peptide inhibitor-based SCI molecules which were identified based on a rational approach involving a ligand–protein binding model.

Analysis of the GSK-3 SCI hits provides important insights into the structural and chemical features required for effective inhibition, and for design of new molecules. First, the existence of a flexible chain bearing a carboxylic acid moiety is mandatory since it strongly interact with the positively charged amino acids (Arg 96, Arg 180, and Lys 205) that bind the “primed” phosphorylated substrates of GSK-3 [36,37]. This novel observation indicates for the first time that a non-phosphorylated group can mimic the “primed” phosphorylation site in GSK-3 substrates [50]. Second, the anthracenone–isoxazole substructure appeared to be the preferred moiety in most of the hit compounds identified. This core forms π–π and cation–π interactions with Phe 93 and occasionally with Arg 96 that are both critically important for substrate binding. Based on these insights, new molecules were designed and synthesized. These showed that replacement of the carboxylic acid with the “native” phosphorylated group and replacing the fluorine at the aromatic ring with electron-withdrawing moieties improved the inhibitory capacity of the compounds. Indeed, the IC_50_ values of the new molecules did not reduce below the one-digit μM range. In our view, this may be an “inherent” feature of SCIs because the kinase–substrate interactions are typically weak to mild interactions, and unlike the ATP binding site, the substrate-binding surface is large and shallow limiting the “complete” burial of a small molecule. From a biological standpoint, this may not necessarily be a disadvantage. This is because cellular concentrations of substrates are far below the μM range, while ATP cellular concentrations are in the μM range. Indeed, studies in cells showed that doses at the 1 μM range and below are sufficient for biological response.

In summary, we presented here novel GSK-3 SCIs as important leads for future drug development. In particular, compounds *4-3* and *4-4* were identified as the most potent inhibitors. Furthermore, the “SCI strategy” described here may be applicable to other protein kinases in generating a new type of protein kinase inhibitor with significant clinical advantages.

## 4. Materials and Methods

### 4.1. Pharmacophore Design and Virtual Screening

A structure-based pharmacophore model, based on the GSK-3-L803F peptide complex [33] was generated using LigandScout 4.0 [51]. The pharmacophore model consisted of six features (F1–F6): two hydrogen bond acceptors (F1, F2), one anionic feature (F3), and three hydrophobic features (F4–F6). Exclusion volumes were also added, based on the protein environment. The initial pharmacophore model was found to be too large to be fitted by drug-like compounds and thus, the pharmacophore feature (F6) corresponding to the hydrophobic interaction with Tyr 216 or Ile 217 was set up as optional. This pharmacophore model was successfully assessed for its ability to recognize the active conformation of L803F peptide and was used to virtually screen a database of ~6.36 million commercially available compounds from the ZINC database [52] with a possibility to purchase molecules from different vendors. Twenty-five conformers were generated for each compound in the database using the OMEGA conformer generator [53] to create a multi-conformer structure database. Screening resulted in 3680 hits, which were subsequently docked into the GSK-3 site. L803F was also included in the docking procedure (and the filtration procedure; see below) to validate the method. Prior to docking, hits were prepared by LigPrep as implemented in Maestro (Schrödinger, USA) at pH = 7 ± 0.2 with the OPLS 2005 force field, including tautomeric variations. Docking was performed using Extra-Precision (XP) Glide [54]. The resulting binding mode were filtered using interaction fingerprints based on the known critical interactions formed between L803F and GSK-3, namely, those involving Arg 96, Arg 180, Lys 205, and Phe 93 [33]. Filtration resulted in 1024 compounds, including the inhibitor L803F. The ten best compounds, as ranked by GlideScore, were selected for purchasing and biological testing. 

For the second round, molecules with 90% similarity to *1-4*, *1-6*, and *1-7* were retrieved from ZINC. The hits (241 analogues for *1-4*, 11 analogues for *1-6*, and 21 analogues for *1-7*), were docked into the GSK-3 substrate binding site and ranked, as before, by GlideScore. Two methods of docking were used: Glide XP and Induced Fit docking (IFD) in which residues within 10 Å of any of the resulting top 20 ligand poses (from the initial docking) were subjected to a conformational search and minimization, whereas residues outside this region were kept fixed. The final 20 new receptor conformations were taken forward for redocking with Glide XP. The binding affinity of each complex was evaluated by GlideScore, and was based on a combination of GlideScore and visual inspection of the resulting binding modes. The third cycle searched for molecules with a substructure composed of an anthracene moiety attached to piperazine/cyclohexane. From this search, 137 compounds were obtained, of which only 15 had a negative charge that could mimic the phosphorylated moiety of L803F and that interacted with the positive pocket of GSK-3 (Arg 96, Arg 180, and Lys 205). Fourteen compounds were commercially available and were taken for further IFD flexible docking for identification of binding mode and for experimental testing. Interaction analysis presented in Table 1 was performed with Maestro (≤2.5 Å for hydrogen or halogen bonds, ≤4.4 Å for π–π stacking interactions and cation—π interaction ≤6.6 Å). 

### 4.2. Chemicals

Compounds selected by virtual screening were purchased from MolPort Riga, Latvia, that supplied compounds from ChemDiv Inc., San Diego, CA USA, Specs, Zoetermeer, The Netherlands, and Enamine Ltd. NJ, USA. The most bioactive compounds *3-7* and *3-8* were purchased in three different batches to validate results. Validation of compounds *3-7* and *3-8* was conducted in house by ^1^H ^13^C NMR, high-resolution mass spectrometry, and HPLC. Data are available upon request. Compounds *4-1*, *4-2*, *4-3*, *4*-4 and *4-5* were synthesized by Wuxi APTech Ltd. Shanghai China. Detailed synthesis description, NMR and Mass spectra and HPLC data are available and can be provided upon request. Compounds were dissolved in DMSO for in vitro studies and with DMSO/1% Tween 80 for studies in cells.

### 4.3. PCA Analysis

A principal component analysis (PCA) was performed for a small subset of all compounds tested in this work together with 29 selected GSK-3-ATP competitive inhibitors retrieved from the CheMBL database (listed in Table S4). Each compound was characterized by a total of 1875 descriptors (1444 1D and 2D descriptors and 431 3D descriptors) calculated by the PaDEL-Descriptor software (http://www.yapcwsoft.com/dd/padeldescriptor/) [55]. The resulting descriptors matrix was submitted to Principal Component Analysis (PCA) as implemented in Canvas (Schrödinger Release 2017-4: Canvas, Schrödinger, LLC, New York, NY, USA, 2017) [56]. The first and second PCs accounted for 81.8% and 7.7% of the original variance, respectively.

### 4.4. In Vitro Kinase Assays

An ELISA-based assay was developed in our laboratory to measure GSK-3 activity. In brief, biotin-labelled IRS-1 peptide substrate [57] was bound to streptavidin-coated 96-well microplates. A GSK-3 assay solution (0.1 μg GSK-3β, 20 mM Tris (pH 7.3), 10 mM MgCl_2_, and 10 μM ATP) was added to each well, together with the candidate compound at indicated concentrations, and the plates were incubated for 15 min at 30 °C. After having been washed (PBS 0.05% tween), the plates were incubated with a specific anti-phospho IRS-1 antibody (Ser^332^) [57] (1:2000), followed by HRP-secondary antibody (1:8000). The signal was developed with TMB solution (abcam, Cambridge UK, Cat# ab210902), stopped with H_2_SO_4_, and monitored in a plate reader (580 nm). Assays were conducted in duplicates in three independent experiments. In the ATP competitive, or, substrate competitive assays, the assays (60 μL) were performed in Eppendorf tubes (to use “unbound” substrate), and 10–20 μL were spotted on the streptavidin-coated plates. Km values were calculated from the non-linear curve fit, and Ki values were calculated by Kapp = Km (1 + [I]/Ki). Bacterially expressed His-tagged rabbit GSK-3β was purified as described [58], and purified human GSK-3α was purchased from abcam (Cat# ab42597). The selectivity of compound *4-4* against a collection of protein kinases was assayed at the International Center for Kinase Profiling at the MRC unit, University of Dundee, UK.

### 4.5. Cell Culture and Cell Extraction

Human Neuroblastoma SH-SY5Y cells were originally provided by American Type Culture Collections. SH-SY5Y cells were grown in RPMI 1640 supplemented with 10% FCS, 5 mM L-glutamine, and 0.5 mg/mL penicillin–streptomycin. The cells were tested for mycoplasma contaminations (Invitrogen, Thermofisher, MA, USA) every 4–6 months. For cytoplasmic preparation, cells were permeabilized with 40 μg/mL digitonin (Sigma, Cat # D5628) in NEH buffer (20 mM Hepes (pH 7.4), 150 mM NaCl, 1 mM EDTA) for 10 min at 4 °C at constant shaking. Cytoplasmic sups (about 800 μL) were collected and subjected to further analyses. For total cell extracts, cells were treated or not with MG132 (abcam, Cat # ab141003), Cells were lysed in ice-cold buffer G (20 mM Tris-HCl, 10% glycerol, 1 mM EDTA, 1 mM EGTA, 0.5% Triton × 100, 0.5 mM orthovanadate, 10 mM β-glycerophosphate, 5 mM sodium pyrophosphate, 50 mM NaF, 1 mM benzaminidine, and protease inhibitors aprotenin, leupeptine, and pepstatin A (25 mg/mL each). Cell extracts were centrifuged at 14,000× *g* for 20 min, and supernatants were collected. Protein concentrations were determined by Bradford assays. For control, cells were treated with the vehicle (DMSO/1% Tween 40) at matched dilutions (1:2000–4000).

### 4.6. Primary Neurons

Hippocampi were isolated from C57BL6/J mice (0–1 day old), and tissues were digested with trypsin type XI (Sigma, Rehovot, Israel, Cat # T1005), and DNase type IV (Sigma, cat #D5025). Cells were suspended in a plating medium including MEM supplemented with 10% FBS, transferrin (0.089 mg/m), GlutaMAX (0.75 U/mL) (Sigma, Cat # 35050-038), insulin (16 μM) (Roche, Basel, Switzerland, Cat # 45865100), and SM1 neuronal supplement (STEMCELL, Seattle, WA, USA, Cat # 05711), and plated on glass coverslips coated with Matrigel (Corning, NY, USA, Cat# 356234) in a 24-well plate. The day after plating and twice a week thereafter half of the medium was removed and was replaced with by fresh feeding medium (plating medium lacking insulin and containing Ara-C (3 μM)) (Sigma, Cat# C6645).

### 4.7. Western Blot Analysis

Equal amounts of proteins (30–50 μg) were subjected to SDS PHAGE gel electrophoresis, transferred to nitrocellulose membranes, and immunoblotted with selected antibodies followed by incubation with HRP-linked anti-rabbit, or, anti-mouse IgG (Cell signaling, MA, USA, and Jackson Immune Research, PA, USA, respectively). ECL developed membranes were imaged in UVITEC Alliance biomolecular imaging, and densitometry analysis of respected bands was analyzed with software provided by the vendor. Representative protein bands were taken from the same gel, and each gel represents three independent experiments. Antibodies used, anti-βcatenin (Transduction Laboratories, Franklin Lakes, NJ, USA, Cat# 610154), anti-phospho-tau (Ser^396^), and anti-GAPDH (Cell Signalin, Cat*#* 9561, 9632, 2118, respectively), anti-βactin (Santa Cruz Biotechnologies, CA, USA, Cat# SC-1615).

### 4.8. Immunostaining

Primary neuron cells were treated with *4-4* (5 μM), CHIR99021 (10 μM) (Merck, MA, USA), or, vehicle (DMSO/1%Tween 80) for 4 hr. Cells were fixed with 4% PFA and immunostained with anti-βcatenin and anti-MAP2 (Sypnactic System, Goettingen, Germany, A neuronal marker) antibodies. βcatenin signal was evaluated by Image J software using co-localization finder plugin (https://imagej.nih.gov/ij/plugins/colocalization-finder.html) that calculated the ratio of βcatenin over MAP2 and DAPI in each cell. The average ratio that was determined for control vehicle-treated cells was set to 1 and respective folds of treated cells were calculated accordingly. 

### 4.9. Image Processing

The gel images shown in the figures were taken from the same experiments.

### 4.10. Statistical Analyses

Statistical analyses were performed with GraphPad Prism 8.2 software. Data are shown as means ± SD of three independent experiments or as indicated in the figure legends. One-way ANOVA with Dunnett’s multiple comparisons, or, unpaired Student’s *t*-test was used for comparison of assays as indicated in the figure legends. *p* < 0.05 was considered significant. 

## 5. Patents

A patent was submitted by Ramot Ltd. at Tel Aviv University.

## Figures and Tables

**Table 1:** GSK-3 interactions with selected compounds. The number of interactions (*), type of interaction, and atom/substructure involved are listed for each GSK-3 residue. Green, H-bond, blue πι–π stacking, black, cation–π, pink, salt bridge.

Name	GSK-3 Interacting Residues					
	Arg 96	Arg 180	Lys 205	Phe 93	Val 214	Others
*1-4*	* Carbonyl of anthracenone * O of CO_2_H	** 2O of CO_2_H	** O of CO_2_H			* Gly 202 with amine at the CO_2_H chain
*1-6*	** thiophene ring Carbonyl of anthracenone, * O of CO_2_H	*O of CO_2_H	*O of CO_2_H			* Glut 97 with phenol hydroxyl and ketone, * Asn 95 with phenol hydroxyl
*1-7*		**** Carbonyl at thiophene ring 2O of CO_2_H O of CO_2_H	* O of CO_2_H	* Bromophenyl ring	* O of CO_2_H	
*2-1*	*** 2O of CO_2_H N of isoxazole	*** 2O of CO_2_H O of CO_2_H	* O of CO_2_H	*** Isoxazole and aromatic rings of anthracenone	* O of CO_2_H	
*2-2*	*** Carbonyl at the anthracenone benzoic acid O of CO_2_H	** O of CO_2_H benzoic acid	** 2O of CO_2_H			* Gly 202 with amine attached to benzoic acid * Lys 85 with O of ether group
*3-7*	*** O of CO_2_H	** 2O of CO_2_H * O of CO_2_H	* O of CO_2_H * O of CO_2_H	** Isoxazole and aromatic rings of anthracenone	* O of CO_2_H	
*3-8*	** 2O of CO_2_H	** 2O of CO_2_H * O of CO_2_H	** O of CO_2_H	** Isoxazole and aromatic rings of anthracenone	* O of CO_2_H	

**Table 2:** GSK-3 interactions with newly designed SCI compounds. The number of interactions (*), type of interaction, and atom/substructure involved are listed for each GSK-3 residue. Green, H-bond, blue π–π stacking, black, cation–π, pink salt bridge. Ionic charges of compounds are indicated.

Name	GSK-3 Interacting Residues							
	Arg 96	Arg 180	Lys 205	Phe 93	Val 214	Phe 67	Lys 85	Ser 66
*4-1*	** O of CO_2_H	** 2O of CO_2_H	* O of CO_2_H	** Isoxazole and aromatic rings of anthracenone	* O of CO_2_H			
*4-2* (-2)	** 2O of PO_3_H_2_ ** aromatic rings of anthracenone	*** 2O of PO_3_H_2_	** 2O of PO_3_H_2_	** Isoxazole and aromatic rings of anthracenone	* O of PO_3_H_2_			
*4-3* (-3)	** N of isoxazole and O of PO_3_H_2_	*** 2O of PO_3_H_2_	*** 2O of P3_4_H_2_	** Isoxazole and aromatic rings of anthracenone	* O of PO_3_H_2_	* O of CO_2_H	** 2O of CO_2_H	
*4-4* *(-2)*	* O of isoxazole, ** aromatic rings of anthracenone, *** 2O of PO_3_H	*** O of PO_3_H_2_ * aromatic rings of anthracenone	** O of PO_4_H_2_	** Isoxazole and aromatic rings of anthracenone	* O of PO_3_H_2_	* Phenol ring		* OH in phen-ol
*4-5*	** O of CO_2_H	*** 2O of CO_2_H	** O of CO_2_H	** Isoxazole and aromatic rings of anthracenone	* O of CO_2_H	** O of CO_2_H at benzoic acid	** 2O of CO_2_H	

## Supplementary Materials

Supplementary Materials can be found at https://www.mdpi.com/1422-0067/21/22/8709/s1. Tables S1–S3: hits identified in the three cycles, Table S4: GSK-3 ATP competitive inhibitors used in PCA analysis.
