# Supplementary material for: Discovery and Design of Novel Small Molecule GSK-3 Inhibitors Targeting the Substrate Binding Site
Source: Int J Mol Sci. 2020 Nov 18;21(22):8709. doi: 10.3390/ijms21228709 (PMC7698860; doi:10.3390/ijms21228709)
Supplement: Supplementary file 1 [file ijms-21-08709-s001.pdf]

SUPPLEMENT

Discovery and Design of Novel Small Molecule GSK-3 Inhibitors Targeting the Substrate Binding Site

Ido Rippin[1], Netaly Khazanov[2], Shirley Ben Joseph[1], Tania Kudinov[1], Eva Berent[1], Sara Melisa Arciniegas Ruiz[1], Daniele Marciano[3], Laura Levy[2], Arie Gruzman[2], Hanoch Senderowitz[2], and Hagit Eldar-Finkelman[1*]

[1] The Department of Human Molecular Genetics & Biochemistry, Sackler School of Medicine, Tel Aviv University, [2]Department of Chemistry, Bar-Ilan University, Ramat Gan, [3]Department of Organic Chemistry, Israel Institute for Biological Research, Ness Ziona , Israel

Contents

Tables S1-S4

**Compound**	**Zinc Number**	**Structure**	**Glide Score (Kcal/mol)**
1-1	ZINC 08438527	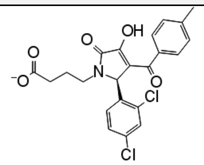	-7.86
1-2	ZINC 17124459	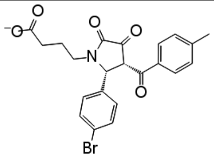	-7.78
1.3	ZINC 19923071	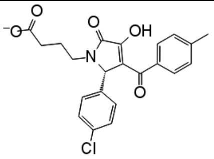	-7.53
1-4	ZINC02457285	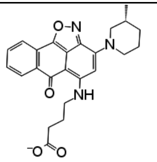	-7.38
1-5	ZINC09509071	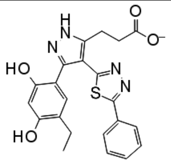	-7.38
1-6	ZINC03426092	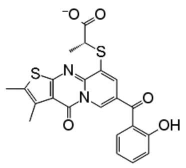	-7.29
1-7	ZINC09763113	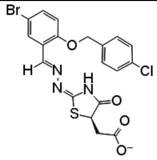	7.19
1-8	ZINC16752220	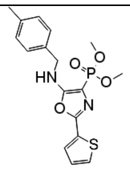	-4.73
1-9	ZINC26842591	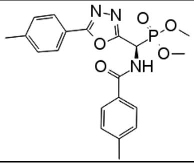	-4.55

1-10	ZINC08915175	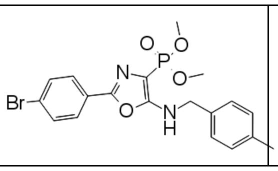	-4.01

Table S1: 1st cycle hits. Identified hits are listed along with their Glide score

**Name**	**Zinc Number**	**Structure**	**Glide Score (Kcal/mol)**	**Related analogue to**
2-1	ZINC03742058	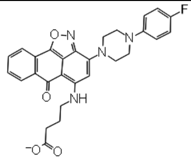	-12.38	*1-4*
2-2	ZINC09780556	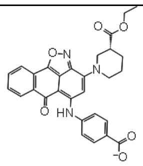	-11.87	*1-4*
2-3	ZINC04128291	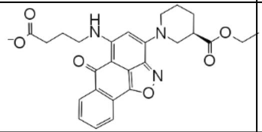	-11.87	*1-4*
2-4	ZINC09780536	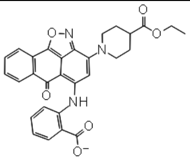	-11.704	*1-4*
2-5	ZINC08722310	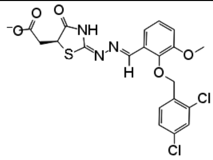	-11.44	*1-4*
2-6	ZINC02457300	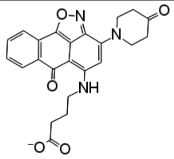	-11.13	*1-4*
2-7	ZINC09763193	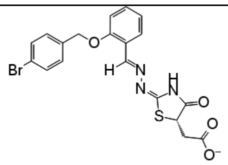	-10.2	*1-6*
2-8	ZINC04128340	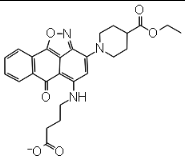	-7.58	*1-4*
2-9	ZINC08722309	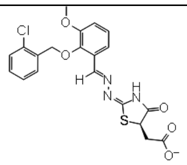	-7.77	*1-7*
2-10	ZINC16115790	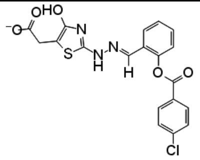	-7.32	*1-7*

2-11	ZINC15857451	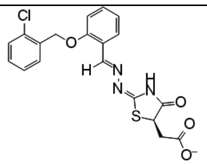	-7.13	*1-7*
2-12	**ZINC02457283**	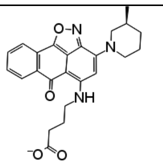		*1-4*

Table S2: 2nd cycle hits. Hits were identified by searching analogues with 90% similarity to compounds *1-4*,*1-6*, and *1-7*. Hits are sorted by score values.

Name	Zinc Number	Structure	Glide Score (Kcal/Mol)
3-1	ZINC04535276	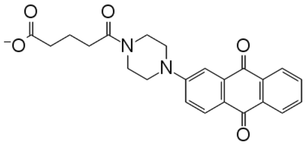	-12.907
3-2	ZINC02457282	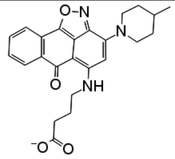	-11.894
3-3	ZINC03742056	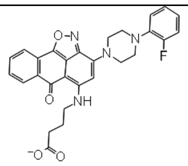	-12.5739
3-4	ZINC02457288	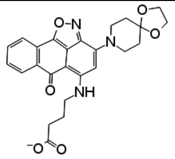	-12.53
3-5	ZINC03742059	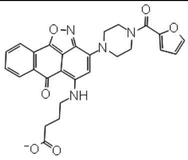	-13.649
3-6	ZINC02457314	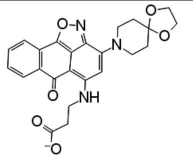	-11.464
3-7	ZINC20108011	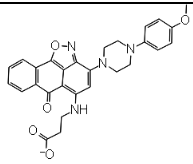	-15.535
3-8	ZINC02494295	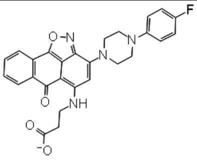	-12.218
3-9	ZINC02494297	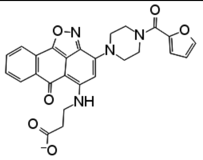	-14.534
3-10	ZINC03958636	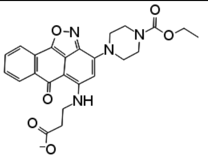	-12.353

3-11	ZINC02457319	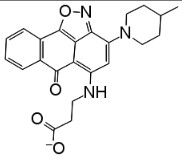	-12.721
3-12	ZINC58012534	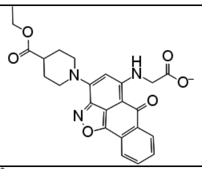	-11.08
3-13	ZINC03742061	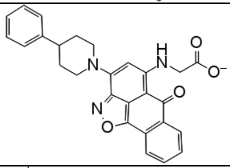	-12.14
3-14	ZINC58012537	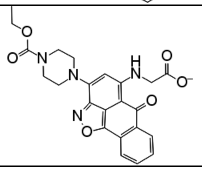	-11.272

Table S3: 3rd cycle hits. The search identified molecules bearing anthracene/piperazine/ cyclohexane substructures and filtered by docking into the GSK-3 substrate binding site.

Table S4: GSK-3 ATP competitive inhibitor used in PCA analysis

**ChEMBL #**	**Name**
CHEMBL102714	SB-216763
CHEMBL322970	SB-415286
CHEMBL1080901	CHIR-99021
CHEMBL259850	AR-A014418
CHEMBL1969664	AZD1080
CHEMBL2178570	AZD4818
CHEMBL405759	TWS119
CHEMBL209849	Indirubin-3-Acetoxime
CHEMBL126077	indirubin-3′-monoxime
CHEMBL336961	1-Azakenpaullone
CHEMBL50894	Alsterpaullone
CHEMBL7463	GF-109203
CHEMBL296586	Kenpaullone
CHEMBL259638	Cazupaullone
CHEMBL409450	6Bio
CHEMBL3747661	VP2.51
CHEMBL362558	LY-2090314
CHEMBL300138	LY317615
CHEMBL2177167	Pyrazine analogue
CHEMBL2182002	Pyrazine analogue
CHEMBL2386102	Melemiede
CHEMBL2163772	dihydroxy-1-aza-9-oxafluorene
CHEMBL319244	Cafeic acid
CHEMBL388978	Staurosporine
CHEMBL50498	Meridianin A
CHEMBL584304	XD-4241
CHEMBL4073799	Dibromocantherelline
CHEMBL75680	Aloisine A
CHEMBL3621636	Hymenidin
